# Prevalence and Correlates of Preschool Overweight and Obesity Amidst the Nutrition Transition: Findings from a National Cross-Sectional Study in Lebanon

**DOI:** 10.3390/nu9030266

**Published:** 2017-03-11

**Authors:** Lara Nasreddine, Nahla Hwalla, Angie Saliba, Christelle Akl, Farah Naja

**Affiliations:** 1 Department of Nutrition and Food Science, Faculty of Agricultural and Food Sciences, American University of Beirut, P.O. Box 11-0236, Riad El Solh, Beirut, Lebanon; ln10@aub.edu.lb (L.N.); nahla@aub.edu.lb (N.H.); aes10@mail.aub.edu (A.S.); cristell@gmail.com (C.A.); 2 Nutrition, Obesity and Related Diseases (NORD), Office of Strategic Health Initiatives, American University of Beirut, P.O. Box 11-0236, Riad El Solh, Beirut, Lebanon

**Keywords:** obesity, preschoolers, prevalence, correlates, diet, socioeconomic status, Lebanon

## Abstract

There is increasing evidence linking early life adiposity to disease risk later in life. This study aims at determining the prevalence and correlates of overweight and obesity among preschoolers in Lebanon. A national cross-sectional survey was conducted amongst 2–5 years old children (*n* = 525). Socio-demographic, lifestyle, dietary, and anthropometric data were obtained. The prevalence of overweight and obesity was estimated at 6.5% and 2.7%, respectively. Based on stepwise logistic regression for the prediction of overweight and obesity (combined), the variance accounted for by the first block (socioeconomic, parental characteristics) was 11.9%, with higher father’s education (OR = 5.31, 95% CI: 1.04–27.26) and the presence of household helper (OR = 2.19, 95% CI: 1.05–4.56) being significant predictors. The second block of variables (eating habits) significantly improved the prediction of overweight/obesity to reach 21%, with eating in front of the television (OR = 1.07, 95% CI: 1.02–1.13) and satiety responsiveness (OR = 0.83, 95% CI: 0.70–0.99) being significantly associated with overweight/obesity. In the third block, fat intake remained a significant predictor of overweight/obesity (OR = 2.31, 95% CI: 1.13–4.75). This study identified specific risk factors for preschool overweight/obesity in Lebanon and characterized children from high socioeconomic backgrounds as important target groups for preventive interventions. These findings may be of significance to other middle-income countries in similar stages of nutrition transition.

## 1. Introduction

Childhood obesity is increasingly recognized as a serious public health concern, with available evidence suggesting a dramatic increase in its worldwide prevalence over the past few decades [1,2]. This increase is documented as early as the preschool years. De Onis et al. [2] showed that the global prevalence of preschool overweight and obesity has escalated from 4.2% in 1990 to 6.7% in 2010, with a projected increase to 9.1% in 2020 [2]. The highest prevalence rates of preschool overweight and obesity in 2010 were reported for the regions of Northern Africa and Western Asia, with an estimate of 17% and 14.7%, respectively [2]. The prevalence of preschool overweight and obesity in these regions, which largely represent the Middle East and North Africa (MENA) region, is projected to rise to over 25% by 2020 [2].

Excess body weight usually results from a complex interaction of genetic, environmental, behavioral and social factors, which may, in concert, modulate the child’s propensity for becoming overweight [3]. Increased food intake, frequent consumption of high‑calorie sweetened beverages and television viewing have been frequently reported as key determinants of the risk of pediatric overweight and obesity [3,4]. Socioeconomic status (SES) has also been identified as an important modulator of the risk of childhood obesity, although discrepancies have been reported in the direction of the relationship between SES and pediatric obesity [5]. In high-income countries, the risk of childhood obesity has been shown to be the highest in lower socioeconomic groups [5], while the opposite was reported for low-income countries [5,6]. Less is known about the relationship between SES and childhood obesity in middle-income countries, particularly those undergoing the nutrition transition [5,6].

Pediatric obesity is associated with adverse physical and psychological effects that may appear in childhood and track into the adult years [7,8,9]. Short-term health consequences of pediatric obesity include metabolic abnormalities such as high blood pressure, dyslipidemia, impaired glucose homeostasis, and metabolic syndrome [7]. In the long term, pediatric obesity tends to persist into adulthood, increasing the risk for obesity-associated morbidities such as cardiovascular disease, type 2 diabetes and some types of cancer [8,9]. Psychologically, obese children commonly suffer from negative body image and low self-esteem [7], that often progress into anxiety and depression in adulthood [8]. Consequently, the early prevention of overweight and obesity is increasingly recognized as a vital strategy to decrease the burden of associated short- and long-term morbidity [10]. The preschool years are identified as an important stage for preventive interventions, as eating patterns established in young childhood tend to track into later life [11].

The development of effective intervention programs aiming at the prevention of pediatric obesity should be based on rigorous investigations of its determinants and associated factors [10]. Most of the studies investigating obesity correlates in preschoolers have been conducted in high-income countries [12] and, as such, findings may not be applicable to low and middle-income countries, where the highest increases in preschool obesity are projected to take place. Among the latter, the Middle East and North Africa (MENA) region has been largely under-represented [12]. In Lebanon, an upper-middle income country of the Eastern Mediterranean basin that is currently undergoing the nutrition transition [13], available evidence documents an increase in obesity prevalence amongst 6–18 years old children and adolescents [14]. However, data on the prevalence and correlates of preschool obesity are lacking. Based on a nationally representative survey, the present study aims at (1) determining the prevalence of overweight and obesity among 2–5 years old preschool children in Lebanon and (2) investigating the association of preschool overweight and obesity with socioeconomic factors, parental characteristics, dietary intakes and eating behavior. Gaining greater insight into factors that are associated with preschool overweight and obesity should orient further studies investigating early life obesity and assist policy makers in setting forth successful culture-specific obesity prevention strategies in the region. The present study undertaken in Lebanon, and particularly the identification of factors that modulate the risk of under-five overweight, may be viewed as a case-study for other middle-income countries in similar stages of the nutrition transition. The study responds to the United Nation’s call for a worldwide commitment to address preschool overweight and reverse its rising trends, as included in the General Assembly’s resolution proclaiming the UN Decade of Action on Nutrition (2016–2025) [15].

## 2. Materials and Methods

### 2.1. Study Population

The data for this study was drawn from the national survey, “Early Life Nutrition and Health in Lebanon”, conducted on a representative sample of Lebanese children (0–5 years) and their mothers. The survey was undertaken between September 2011 and August 2012. A stratified cluster sampling strategy was followed, whereby the strata were the six Lebanese governorates and the clusters were selected further at the level of districts. Within each district, households were selected following a probability proportional to size approach, whereby a higher number of participating households were drawn from more populous districts. The selection of the households was carried out using systematic sampling. Housing units constituted the primary sampling units in the different districts of Lebanon. To participate in the study, the household ought to include a mother and a child below five years of age. The child had to be Lebanese, born at term (of gestational age at birth ≥37 weeks), not suffering from any chronic illness, inborn errors of metabolism or physical malformations that may interfere with his/her feeding patterns and body composition, and not reported as being ill during the past 24 h (i.e., on the day that would be recalled for food intake). Of the 1194 eligible households that were contacted, 1029 participated in the survey (response rate 86%). The main reasons for refusal were time constraint, child being sick, lack of husband’s consent or disinterest in the study. In face to face interviews with participating mothers, trained nutritionists collected data, using age-specific multi-component questionnaires covering information on demographic, socioeconomic, eating habits and dietary intakes. Anthropometric measurements were obtained from both mother and child. Dietary intake was assessed using the United States Department of Agriculture (USDA) multiple pass 24-h recall (24-HR) [16]. Interviews were held in the household setting and lasted for approximately one hour. Quality control measures including pre-testing of the study instruments, equipment and data collection procedure in addition to training and field monitoring, were applied. All questionnaires were designed by a panel of experts including scientists in the fields of epidemiology and nutrition and were tested on a convenience sample of 100 households to check for clarity and cultural sensitivity.

### 2.2. Ethical Considerations

The design and conduct of the survey were performed according to the guidelines laid down in the Declaration of Helsinki, and all procedures involving human subjects were approved by the Institutional Review Board of the American University of Beirut. A written informed consent was obtained from all mothers prior to participation.

### 2.3. Data Collection

For the purpose of this study, data of children aged between 2 and 5 years were used (*n* = 531). The availability of this sample size allowed the estimation of a 10% prevalence of overweight and obesity at a 95% confidence interval and a precision of ±2.5% [17]. Survey data used in this study included demographic, socioeconomic, and parental characteristics, eating habits, anthropometric measurements, as well as dietary intakes. Demographic characteristics consisted of the following: sex of the child, age of the child and the mother (in years), mother’s marital status (married, not married) and number of children in the family; socioeconomic indicators included father’s and mother’s education levels (primary or less, intermediate, high school, and above) and household’s monthly income, which are the most commonly utilized indicators of socioeconomic status [18], in addition to mother’s employment (working, not working), presence of paid household helper, and household crowding index. Moreover, given that the formal age at which children enroll in the preprimary level of schools is three years in Lebanon [19], information on the type of school the child attends (private vs. public) was also obtained as one of the socioeconomic indicators. In fact, in Lebanon, there exist strong social inequalities among those attending private and public schools, with the private schools enrolling the highest proportion of students from a high SES, and the public schools enrolling those from a low SES [19,20]. The assessment of the child’s eating habits included weekly frequency of breakfast consumption, eating in front of the television (TV), eating out, and eating the same meal with the family. In addition, early life feeding practices were assessed by the mother’s retrospective recall of breastfeeding duration, age of introduction of formula and of solid food. The child satiety responsiveness and food responsiveness were evaluated using questions derived from the Child Eating Behaviour Questionnaire (CEBQ) [21].

### 2.4. Anthropometric Assessment

Information on birth weight of the child was obtained from the mother. Anthropometric measurements were performed, including weight and height of both mothers and children. Participants were weighed to the nearest 0.1 kg in light indoor clothing and with bare feet or stockings, using a standard clinical balance (Seca, model 770, Hamburg, Germany). Measurements of the weight were taken twice and repeated a third time if the first two measurements differed by more than 0.3 kg. Using a portable stadiometer (Seca, model 213, Hamburg, Germany) height was measured without shoes. Measurements of the height were taken twice and repeated a third time if the first two measurements differ by more than 0.5 cm. The average values of weight and height were used for the calculation of BMI, which is computed as the ratio of weight (kilograms) to the square of height (meters). Weight and height were not collected from women who were pregnant at the time of the interview (*n* = 42), due to the limitations of BMI use during pregnancy.

Overweight and obesity among mothers were assessed using the World Health Organization (WHO) criteria for body mass index (BMI) [22]. For children, the prevalence of overweight and obesity was assessed using the WHO-2006 criteria based on sex and age specific BMI *z*-scores, which were calculated using the WHO AnthroPlus software (WHO 2009, Department of Nutrition for Health and Development, Switzerland, Geneva) [23,24]. Accordingly, the following cutoffs were adopted: +1 < BMI-for-age *z*-score ≤ +2 (at risk of overweight), +2 < BMI-for-age *z*-score ≤ +3 (overweight), and BMI-for-age *z*-score > +3 (obesity). In addition, and in order to allow for comparability with other studies, the prevalence of overweight and obesity was assessed using two other definitions, including the Center for Disease Control and Prevention-2000 (CDC-2000) and International Obesity Task Force (IOTF):

1. According to the CDC 2000 reference [25,26] cut-off values were defined based on sex and age specific BMI percentiles: 85th to <95th percentile (overweight) and ≥95th percentile (obesity).
2. According to the IOTF standard [27], overweight and obesity were based on centiles passing at age 18 years through BMI 25 kg/m^2^ and 30 kg/m^2^, respectively.

### 2.5. Dietary Intake of Children

Dietary intake of children was assessed by a single multiple pass 24 HR. Even though various methods have been developed for the assessment of dietary consumption, including dietary recalls, food frequency questionnaires (FFQs) and food records [28], the choice of the 24 HR approach in this study may be explained by (1) the lack of validated culture-specific FFQs targeting under-five children in Lebanon and the MENA region; and (2) the practical difficulties of using food records in the context of national surveys, given the burden that this method may impose on participants and given its literacy requirements [28]. In addition, acknowledging the practical challenges of administering repeated multiple 24 HRs in the context of a national survey and the impact that this approach may have on response rate [29], we have opted to use a single 24 HR, with mothers serving as the main proxy. In case another caretaker shared the responsibility of feeding the child, the mother directly consulted with him/her for additional information pertinent to the dietary interview. The 24-HRs were carried using the multiple pass food recall five-step approach, developed by the USDA [16]. This approach has consistently showed attenuation in the 24-HRs’ limitations [30]. The steps followed included (1) quick food list recall; (2) forgotten food list probe; (3) time and occasion at which foods were consumed; (4) detailed overall cycle; and (5) a final probe review of the foods consumed. While collecting the dietary data, specific reference was made to solicit information about food that were consumed at daycare or school. The Nutritionist Pro software (version 5.1.0, 2014, First Data Bank, Nutritionist Pro, Axxya Systems, San Bruno, CA, USA) was used for the analysis of the dietary intake data and to estimate energy and macronutrients’ intakes. For composite and mixed dishes, standardized recipes were added to the Nutritionist Pro software using single food items. Within the Nutritionist Pro, the USDA database was selected for analysis (SR 24, published September 2011). Food composition of specific Lebanese foods (not included in the Nutritionist Pro software database) was obtained from food composition tables for use in the Middle East [31].

### 2.6. Data Analysis

Frequencies and percentages, as well as means and standard deviations (SD), were used to describe categorical and continuous variables, respectively. Crowding index was calculated as the total number of co-residents per household divided by the total number of rooms, excluding the kitchen and bathrooms. Bivariate logistic regression was used to examine the sociodemographic, eating habits and dietary intake correlates of overweight and obesity in the study population. In this regression, the dependent variable was ‘overweight/obesity’, as defined by the WHO-2006 (BMI-for-age *z*-score > +2) [23]. In order to examine the independent effect of each group of variables (sociodemographic and parental characteristics, eating habits and dietary intake) in predicting overweight and obesity, a stepwise logistic regression was conducted. Block 1 consisted of sociodemographic and parental characteristics, block 2 included eating habits variables and block 3 was comprised of dietary intake data. Variables chosen to be included in the multivariate modeling were either significant in the bi-variate analysis and/or were important according to the literature. Data analysis was carried out using Statistical Package for Social Sciences 22.0 (SPSS for Windows, 2013, SPSS Inc., Chicago, IL, USA). A *p*-value less than 0.05 was considered statistically significant.

## 3. Results

Out of the 531 survey participants, six children had incomplete sociodemographic and dietary data and, hence, were excluded from the remaining analyses and results. Among children participating in this study (*n* = 525), prevalence rates of overweight and obesity using the four definitions are presented in Table 1. According to the WHO-2006 criteria, prevalence estimates of overweight and obesity were 6.5% and 2.7%, respectively. Higher estimates were obtained using IOTF and CDC-2000 reference cutoffs, with the latter presenting the highest estimates (overweight 16.1% and obesity 10.6%). Although the WHO-2006 presented lowest estimates of overweight and obesity compared to other references, it also had the lowest prevalence of normal weight (64.6%) since, according to this reference, a proportion of normal weight are grouped under a distinct category called ‘at risk of overweight’, with a prevalence of 26.3%. This particular category does not exist within the other references. No significant difference in overweight or obesity rates were observed between boys and girls (Table 1).

Table 2 presents descriptive statistics for the sociodemographic, socioeconomic, and parental characteristics, eating habits, and dietary intake of study participants, in addition to the univariate associations of these correlates with overweight (including obesity) (BMI-for-age *z*-score > +2). The mean age of preschoolers was of 3.3 ± 0.87 years, with 53.5% boys and 46.5% girls. Most parents had intermediate level education (62.8% of fathers and 61.5% of mothers). The majority of mothers did not work (85.1%) and most of the households did not have a household helper (84.1%). Average maternal BMI was estimated at 26.71 ± 5.18 kg/m^2^ (Table 2), with the prevalence of overweight and obesity being estimated at 58.4% among mothers (34.3% overweight and 24.1% obese) (data not shown). When looking at early life feeding practices, almost half of the participating preschoolers were breastfed for less than six months (47.8%), 16.8% for 6–11 months (data not shown) and 35.4% for more than 12 months. The average breastfeeding duration was estimated at 8.9 ± 8.7 months, while the average age of formula milk introduction and of solid food introduction were estimated at 1.3 ± 1.7 and 5.8 ± 2.49 months, respectively. In the study sample, the average weekly frequency of eating breakfast was of 6.7 ± 1.6, while the mean frequencies of “eating in front of the TV” and of “eating the same meal as the family” were estimated at 4.8 ± 6 and 10.7 ± 6.2, respectively. As for dietary intake variables, energy and macronutrient consumption were categorized as above or below the respective median values. These median values corresponded to 1509 kcal/day for energy, 39.3% for “energy consumption from fat”, 48.6% for “energy consumption from CHO”, and 13.15% for energy consumption from protein. As shown in Table 2, lower odds of overweight/obesity were found in families with number of children ≥3, attending a public school, a crowding index ≥1, longer duration of breastfeeding (≥12 months), eating the same meal with the family at home and a higher satiety responsiveness. The odds of overweight/obesity increased significantly with father’s education level, mother’s education level, presence of paid helper at home, income higher than 1,500,000 Lebanese lira (LL), mother’s BMI, higher frequency of eating while watching TV, eating out, and a greater food responsiveness. Among dietary intake variables, energy consumption from fat was associated with a higher odd of overweight/obesity (Table 2).

The results of the stepwise logistic regression examining the independent effects of socioeconomic and parental characteristics, eating habits, as well as dietary intakes on the odds of overweight/obesity are presented in Table 3. The variance accounted for by the first block (socioeconomic and parental characteristics) was 11.9%. Within this block, father’s education, mother’s BMI, presence of a paid helper, and crowding index made significant contributions (*p* < 0.05) to the prediction of overweight/obesity among study participants. The second block of variables was related to early life feeding and eating habits, including breastfeeding duration, eating while watching TV, eating out, eating the same meal with the family at home, satiety responsiveness, and food responsiveness. After controlling for the socioeconomic and parental characteristics, these variables significantly improved the prediction of overweight/obesity to reach 21% (*p* < 0.01). Eating in front of the TV was associated with an 8% increase in the odds of overweight/obesity (OR: 1.08, 95% CI: 1.02–1.1), while a higher score of satiety responsiveness was associated with lower odds of overweight/obesity in the study population (OR: 0.8, 95% CI: 0.68–0.99). As for the third block (dietary intakes), energy consumption from fat remained a significant predictor of preschool overweight/obesity, after adjusting for other variables (OR: 2.31, 95% CI: 1.13–4.75). (Table 3).

Given the association between preschool overweight/obesity and fat intake, additional analyses were conducted to assess the major food contributors to fat and energy intakes in the study sample. The results showed that, besides milk and dairy products which appeared as the largest contributor to fat intake (24.4%), the main sources of fat were fast food and salty snacks (21.6%), followed by beef, poultry, and eggs (12.3%), rice and rice-based dishes (8.7%), and sweet deserts (8.2%). Similarly, the main contributor to daily energy intake was milk and dairy products (17.6%), followed by fast food and salty snacks (16.2%), breads (12.5%), sweets (9.6%), meat, poultry, and eggs (9.04%), rice and rice-based dishes (7.8%), and sweetened beverages (6.1%) (data not shown).

## 4. Discussion

This paper reports on the national prevalence of overweight and obesity in Lebanese 2–5 years old preschoolers and provides evidence linking specific socioeconomic, dietary, and lifestyle factors to increased risk of overweight and obesity in this age group. In view of the scarcity of data on the determinants of childhood obesity in the MENA, the present study’s findings may be viewed as a case-study for other middle-income countries of the region, in similar stages of the nutrition transition.

Using the WHO-2006 BMI criteria, findings of this study show that the prevalence of overweight/obesity combined (BMI-for-age *z*-score > +2) (9.1%) amongst Lebanese preschoolers exceeds the global prevalence estimate of preschool overweight/obesity for 2010 (6.7%), as well as the estimate reported for developing countries (6.1%) [2]. The prevalence rates of overweight (6.4% in boys and 6.6% in girls) and obesity (2.8% in boys and 2.5% in girls) amongst Lebanese preschoolers are similar to those reported from several European countries, while being lower than those reported from some other MENA countries such as Bahrain and Qatar [33,34,35,36,37,38,39,40,41,42,43,44]. (Table 4). To allow for a comparison with findings reported from other countries, data were re-analyzed according to the IOTF and CDC criteria. Based on the IOTF criteria, current prevalence estimates of overweight (12.9% in boys and 13.9% in girls) and obesity (2.9% in boys and 4.9% in girls) amongst Lebanese preschoolers are within the range reported from developed countries such as Australia and Canada [33,44,45,46]. When using the CDC criteria, the prevalence estimates of preschool overweight (14.7% in boys and 17.6% in girls) and obesity (11.1% in boys and 10.2% in girls) in Lebanon are found to be higher than those reported from Iran (overweight: 9.8 and 10.3% respectively; obesity: 4.8 and 4.5% respectively) [47], with the prevalence of obesity being also higher than that reported from the United States of America [48].

Pediatric obesity and excess body weight often result from a complex interaction between genetic and lifestyle factors [10]. Our finding of a positive significant association between preschool overweight/obesity and maternal BMI corroborates those reported from other studies and underscores the importance of genetic factors in the etiology of body fatness [10,50]. Our study’s findings also underscore the importance of socioeconomic and lifestyle factors in modulating the risk of pediatric overweight. To our knowledge, this study is the first from the MENA region to investigate and document a positive association between preschool overweight/obesity and SES as assessed by several indicators, including type of school attended, father’s educational level, mother’s educational level, presence of a paid helper, crowding index, and monthly income. Socioeconomic and parental characteristics made the highest contributions to the prediction of overweight/obesity among study participants, accounting for 12% of the model variance. Previous studies conducted in other parts of the world suggest that SES affects the risk of developing obesity in children, but available evidence highlights disparities in the relationship between SES and pediatric obesity in industrialized vs. developing countries [51]. While children from low SES groups are at higher risk of obesity in industrialized countries, pediatric obesity appears to be predominantly a problem of the rich in low-income countries [18,51]. Less is, however, known about the relationship between SES and childhood obesity in middle-income countries, particularly those undergoing the nutrition transition [5,6]. The present study showed that, in Lebanon, a middle-income country undergoing the nutrition transition, the odds of preschool overweight/obesity were positively associated with SES. In fact, higher paternal education, which is one of the most commonly adopted SES indicators [18], was associated with a five-fold increase in the odds of preschool overweight/obesity, and a higher crowding index, which reflects a lower SES, was associated with lower odds of overweight/obesity in this age group. These findings are in agreement with those reported from several developing countries [52,53] and highlight the role of upward mobility and SES in modulating the family’s economic and cultural resources, all of which may bear ramifications on lifestyle and, therefore, obesity risk in childhood. The observed positive association between SES and preschool overweight/obesity in Lebanon may be explained by the fact that children from affluent families may have higher access to energy-rich diets and electronic games as well as more opportunities for eating out, putting them at a higher risk for positive energy balance and weight gain [5]. Additional analyses conducted in this study have in fact shown significant associations between “eating out”, “eating the same meal with the family”, and main drivers of SES, such as paternal education (data not shown).

Of interest, the study findings showed that the presence of a paid helper in the household was associated with a two-fold increase in the odds of overweight/obesity in Lebanese preschoolers, even after adjustment for other SES indicators including father’s education and crowding index. It is important to note that in the Lebanese context, the responsibility of feeding the child is often shared with the household helper, and this type of child care is becoming increasingly common in the country. Available estimates suggest that, in 2010, Lebanon hosted 117,941 paid sleep-in domestic workers who come from foreign countries, including the Philippines, Sri Lanka, Ethiopia, and Bangladesh, and who live in their employer’s house for the duration of their contract [54]. Our findings of a positive association between preschool overweight/obesity and the presence of a household helper echo those reported by a population-based birth cohort of Chinese children, where “informal” non-parental child care at each of 3, 5, or 11 years of age was independently associated with higher BMI-for-age *z*-scores and with the presence of childhood overweight levels [55]. Our results are also in agreement with findings stemming from Western societies, where several studies [56,57,58] have reported an association between pediatric obesity and “informal” rather than parental child care [55]. Needless to say that caregivers, including household helpers, may play an important role in influencing the child’s dietary practices and eating habits [59]. While parents may play a more active role in supervising the child’s eating behavior, household helpers, who are usually hired for housework as well as child care, may not be able to spend much time and effort on enforcing dietary recommendations, limiting the child’s consumption of energy-dense favorite foods or restraining TV viewing [55]. In our study, the time spent on TV viewing was not directly assessed, but a positive association was found between preschool overweight/obesity and eating while watching TV. Several studies have shown that eating in front of the TV is positively associated with higher BMI among children, an association that is independent of the overall time spent watching TV or the sedentary behavior that accompanies it [60,61]. Dubois et al. showed that four- to five-year-old children who frequently ate in front of the TV had higher BMI relative to their peers, while no significant associations were found between the child’s BMI and the overall time spent watching TV [3]. There are several mechanisms that could link preschool obesity to the act of eating while watching TV. First, children who eat in front of the TV may miss out on the nutritional and psychosocial benefits of family meals [3,61]. In addition, eating in front of the TV is associated with increased exposure to the advertisements of unhealthy foods at meal time hours and with mindless eating that often results in the consumption of larger food portions [62]. Available evidence suggests that children who are given the opportunity to eat while watching TV may become less sensitive to internal cues of satiety [63]. In this study, higher satiety responsiveness was associated with significantly lower odds of overweight/obesity in preschoolers. These findings are in agreement with previously reported inverse association between satiety response and preschoolers’ BMI [64,65].

The results of the present study showed that higher dietary fat intake was associated with a two-fold increase in the prevalence of preschool overweight/obesity. Even though the evidence in the literature is inconsistent, several studies have shown that percentage energy intake from fat was greater in obese children compared with their non-obese counterparts, although total energy intake was not different, a finding that is in agreement with the results of the present study [66,67,68]. Other studies using BMI and or skinfold measures to estimate adiposity have documented a positive association between the percentage of energy intake from fat and body fatness in children before and after controlling for maternal BMI, a finding that is also in agreement with the results of the present study [69,70]. There are a number of mechanisms through which dietary fat may play a role in the development of overweight and obesity. Compared to protein and carbohydrate, fat is more palatable and energy dense, has less ability to regulate hunger and satiety and, hence, is more likely to lead to passive over-consumption [69,71,72]. In addition, and in contrast to protein and carbohydrate, which have comparatively limited storage capacity and are therefore preferentially oxidized when energy intake exceeds expenditure, there is no regulation of fat balance or limit on storage of excess energy from fat, making it more efficiently (about 96%) stored than excess carbohydrate energy (60% ± 80%) [66,73]. Thus, given the poor regulation of fat at both the levels of consumption and oxidation, a chronically high fat diet may compromise the regulation of energy balance and lead to weight gain [71,72]. The study findings may thus call for dietary intervention strategies aiming at reducing fat intake amongst preschoolers in Lebanon [74]. These interventions should, at least partly, focus on the observed main sources of fat in this age group, which included fast food, salty snacks, and sweets.

The strengths of the study include the national design of the survey, the use of a culture and population specific questionnaire in data collection and the measurements of anthropometric characteristics instead of self-reporting. In addition, several indicators were used in this study for the assessment of SES, all of which have converged in documenting a positive association with preschool overweight/obesity in Lebanon. The results of this study should, however, be considered in light of the following limitations. Though every effort was exerted in order to ensure the representativeness of the sample, a comparison of the study sample distribution across governorates with that of the Lebanese population for the same age group showed a few discrepancies. For instance, while South Lebanon constitutes 21.1% of the population, this percentage was only 16.8% in the study population. This difference was compensated by a higher representation of Mount Lebanon (32% in the study sample vs. 28.8% in the population), and Beirut (10.5% in the study sample vs. 7.7% in the population). Such discrepancies resulted from the fact that the research team faced security clearance challenges in South Lebanon, whereby access to this governorate is controlled by tight security measures. In our study, dietary information was based on the collection of one 24-HR, which may not be representative of dietary intakes at the individual level. However, despite its well-known limitations, such as reliance on memory and day-to-day variation, the 24-HR may provide accurate estimates of energy intake at the population level [75]. In the present study, dietary information was collected by the multiple pass 24-HR approach, which was shown to provide accurate estimates of dietary intake in children [76]. In addition, the recalls were taken by research nutritionists who went through extensive training prior to data collection in order to minimize interviewer errors. Similarly, inter-observer measurement error in anthropometric assessment was minimized by extensive training and follow up to maintain quality of measurement among all research nutritionists. It is important to note that physical activity was not assessed in the present study, and as such its association with preschool overweight/obesity was not investigated. However, variability in physical activity tends to be rather limited in this age group and engagement in structured exercise is quite uncommon [77,78]. It is important to note that no information was available regarding whether the participating mothers have recently delivered and/or are currently breastfeeding at the time of the interview. For these two groups of mothers, given that BMI may not be reflective of their usual weight status, their inclusion in the analysis may have attenuated the results found in this study. Similarly, data on access to non-traditional food markets, body image of children, means of transportation and the built environment, which may play an important role in influencing the risk of childhood obesity [79,80] were not collected in this study. Finally, the cross-sectional design of the study allows us to test associations rather than to assess any causal relationships.

## 5. Conclusions

This study showed that the rates of overweight and obesity amongst Lebanese preschoolers exceed the global prevalence estimate of preschool overweight/obesity, as well as the estimate reported for developing countries [2]. This study has also provided the first evidence from the MENA region on the link between preschool overweight/obesity and higher SES, thus, potentially serving as a case-study for other middle-income countries in similar stages of the nutrition transition. In addition, specific dietary behaviors, including eating while watching TV and consuming a high fat diet, were shown to be associated with increased risk of overweight and obesity in Lebanese preschoolers, which corroborate findings stemming from previous studies on this age group. Taken together, the study’s findings highlight the importance of the home environment in modulating the young child’s lifestyle and dietary habits and hence obesity risk early in life. In this context, the results of the study call for education interventions aiming at raising parental awareness on preschool overweight in Lebanon, a country where early life “chubbiness” may not be perceived as a health threat but is rather culturally believed to be a sign of good health and an inherent component of the child’s “cuteness”.

Recognizing that the development of early life obesity prevention strategies should rely on evidence-based public health approaches, the results of this paper could represent a stepping stone for the formulation of effective interventions and policies aiming at curbing the epidemic of pediatric obesity in Lebanon. Family-focused interventions and behavioral strategies, coupled with school-based interventions and policies, are needed to instill healthy lifestyle and dietary habits early in life [6].

## Figures and Tables

**Table 1:** Overweight/obesity prevalence among 2–5 years old Lebanese preschoolers, according to WHO-2006, IOTF and CDC-2000 criteria.

Weight Status	Total (*n* = 525)	Boys (*n* = 281)	Girls (*n* = 244)
	*n* (%)		
**WHO-2006 reference ^a^**			
**Normal weight ^b^**	339 (64.6)	177 (63.0)	162 (66.4)
**At risk of overweight**	138 (26.3)	78 (27.8)	60 (24.6)
**Overweight ^c^**	34 (6.5)	18 (6.4)	16 (6.6)
**Obese**	14 (2.7)	8 (2.8)	6 (2.5)
**Overweight and obese**	48 (9.1)	26 (9.3)	22 (9.0)
**IOTF reference ^d^**			
**Normal weight ^b^**	433 (82.8)	235 (84.2)	198 (81.1)
**Overweight ^c^**	70 (13.4)	36 (12.9)	34 (13.9)
**Obese**	20 (3.8)	8 (2.9)	12 (4.9)
**Overweight and obese**	90 (17.2)	44 (15.8)	46 (18.8)
**CDC-2000 reference ^e^**			
**Normal weight ^b^**	383 (73.1)	207 (74.2)	176 (72.1)
**Overweight ^c^**	84 (16.1)	41 (14.7)	43 (17.6)
**Obese**	56 (10.6)	31 (11.1)	25 (10.2)
**Overweight and obese**	140 (26.8)	72 (25.8)	68 (27.9)

No significant differences between genders were observed. ^a^ World Health Organization-2006 reference [23]; ^b^ The normal weight category included thinness, with only one child identified as thin based on the WHO 2006 criteria [23], 18 based on IOTF criteria [32], and 16 based on CDC [25,26]; ^c^ “Overweight” category does not include “Obese”; ^d^ International Obesity Task Force reference [27]; ^e^ Center for Disease Control and Prevention-2000 reference [25,26].

**Table 2:** Association of demographic, socioeconomic, eating habits and dietary intakes with preschoolers’ weight status, Lebanon (*n* = 525) ^a^.

Variables		Children Adiposity Status ^c^			
	Total ^b^ (*n* = 525)	Normal Weight (Including at Risk of Overweight) (*n* = 477)	Overweight (Not Including Obese) (*n* = 34)	Obese (*n* = 14)	Univariate Analysis ^d^
	Demographic, socioeconomic and parental characteristics *n* (%)				OR [95% CI]
**Gender**					
Boys	281 (53.5)	255 (53.5)	18 (52.9)	8 (57.1)	1 [ref]
Girls	244 (46.5)	222 (46.5)	16 (47.1)	6 (42.9)	0.97 [0.53–1.76]
**Child’s Age (years)**					
mean ± SD	3.32 + 0.87	3.32 + 0.88	3.33 + 0.78	3.53 + 0.57	1.08 [0.77–1.53]
**Mother’s Age (years)**					
mean ± SD	32.78 + 5.97	32.68 + 5.92	33.11 + 6.49	35.21 + 6.27	1.02 [0.98–1.08]
**Mother’s marital status**					
Married	514 (97.9)	468 (98.1)	33 (97.1)	13 (92.9)	1 [ref]
Unmarried (divorced or widowed)	11 (2.1)	9 (1.9)	1 (2.9)	1 (7.1)	2.26 [0.47–10.77]
**Number of children in the family**					
≤2 children	272 (51.8)	236 (49.5)	26 (76.5)	10 (71.4)	1 [ref]
≥3 children	253 (48.2)	241 (50.5)	8 (23.5)	4 (28.6)	**0.32 [0.16–0.64]**
**Type of school attended ^e^**					
Private	310 (74.5)	274 (72.9)	25 (89.3)	11 (91.7)	1 [ref]
Public	106 (25.5)	102 (27.1)	3 (10.7)	1 (8.3)	**0.29 [0.10–0.86]**
**Father’s education**					
Primary or less	116 (22.1)	114 (24.3)	2 (6.1)	0 (0.0)	1 [ref]
Intermediate	324 (62.8)	290 (61.8)	26 (78.8)	8 (57.1)	**6.68** [1.57–28.27]
High school and above	76 (14.7)	65 (13.9)	5 (15.2)	6 (42.9)	**9.64** [2.07–44.86]
**Mother’s education**					
Primary or less	101 (19.2)	98 (20.5)	2 (5.9)	1 (7.1)	1 [ref]
Intermediate	323 (61.5)	292 (61.2)	23 (67.6)	8 (57.1)	**3.46** [1.03–11.59]
High school and above	101 (19.2)	87 (18.2)	9 (26.5)	5 (35.7)	**5.25** [1.46–18.90]
**Mother’s employment**					
Working	78 (14.9)	68 (14.3)	5 (14.7)	5 (35.7)	1 [ref]
Not Working	447 (85.1)	409 (85.7)	29 (85.3)	9 (64.3)	0.63 [0.30–1.32]
**Presence of paid helper**					
No	439 (84.1)	406 (85.7)	25 (73.5)	8 (57.1)	1 [ref]
Yes	83 (15.9)	68 (14.3)	9 (26.5)	6 (42.9)	**2.71 [1.40–5.26]**
**Crowding index**					
<1 person/room	60 (11.5)	50 (10.5)	6 (17.6)	4 (28.6)	1 [ref]
≥1 person/room	464 (88.5)	426 (89.5)	28 (82.4)	10 (71.4)	**0.45 [0.21–0.96]**
**Monthly income**					
Low (<1,000,000 LL)	172 (39.3)	161 (40.6)	10 (33.3)	1 (9.1)	1 [ref]
Medium (1,000,000–1,500,000 LL)	108 (24.7)	100 (25.2)	8 (26.7)	0 (0.0)	1.17 [0.45–3.01]
High (>1,500,000 LL)	158 (36.1)	136 (34.3)	12 (40.0)	10 (90.9)	**2.36 [1.10–5.05]**
Mother’s BMI (Kg/m^2^) *	26.71 ± 5.18	26.59 ± 5.17	27.99 ± 5.63	27.51 ± 4.40	**1.05 [1.00–1.10]**
	**Breastfeeding history and eating habits**				**OR [95% CI]**
**Breastfeeding duration**					
<12 months	339 (64.6)	300 (62.9)	26 (76.5)	13 (92.9)	**1 [ref]**
≥12 months	186 (35.4)	177 (37.1)	8 (23.5)	1 (7.1)	**0.39 [0.18–0.82]**
**Breastfeeding duration (in months)**	8.94 ± 8.73	9.11 ± 8.92	8.10 ± 6.84	4.92 ± 3.85	1 [ref]
					0.97 [0.94–1.01]
**Age of formula/cow’s milk’s introduction (months)**	1.34 ± 1.71	1.34 ± 1.70	1.66 ± 2.07	0.70 ± 0.89	1 [ref]
					0.99 [0.78–1.27]
**Age of solid food’s introduction (months)**	5.80 ± 2.49	5.75 ± 2.51	6.48 ± 2.57	5.89 ± 1.47	1 [ref]
					1.07 [0.98–1.18]
**Child’s birth weight (kg)**	3.19 ± 0.55	3.18 ± 0.56	3.30 ± 0.52	3.32 ± 0.49	1.00 [1.00–1.01]
**Eating breakfast (weekly frequency)**	6.74 ± 1.64	6.75 ± 1.68	6.88 ± 0.53	6.21 ± 2.00	0.97 [0.81–1.16]
**Eating in front of the TV (weekly frequency)**	4.81 ± 6.00	4.61 ± 5.59	6.67 ± 9.87	7.14 ± 6.58	**1.04 [1.01–1.09]**
**Eating out (weekly frequency)**	0.40 ± 0.87	0.38 ± 0.78	0.70 ± 1.65	0.57 ± 0.82	**1.27 [1.01–1.62]**
**Eating the same meal as the family at home (weekly frequency)**	10.74 ± 6.24	10.92 ± 6.29	9.55 ± 5.55	7.60 ± 5.54	**0.94 [0.89–0.99]**
**Satiety responsiveness**	8.56 ± 2.15	8.63 ± 2.15	7.94 ± 2.02	7.71 ± 2.05	**0.84 [0.73–0.97]**
**Food responsiveness**	3.87 ± 1.39	3.82 ± 1.37	3.97 ± 1.50	5.14 ± 1.46	**1.28 [1.03–1.58]**
	**Dietary intake (per day) ^f^*n* (%)**				**OR [95% CI]**
**Total energy (Kcal)**					
Below the median ^f^	258 (50.1)	234 (50.1)	18 (52.9)	61 (42.9)	1 [ref]
Above the median ^f^	257 (49.9)	233 (49.9)	16 (47.1)	8 (57.1)	1.00 [0.55–1.81]
**Energy consumption from fat (%)**					
Below the median ^f^	259 (50.3)	243 (52.0)	11 (32.4)	5 (35.7)	1 [ref]
Above the median ^f^	256 (49.7)	224 (48.0)	23 (67.6)	9 (64.3)	**2.17 [1.15–4.06]**
**Energy consumption from CHO (%)**					
Below the median ^f^	257 (49.9)	227 (48.6)	22 (64.7)	8 (57.1)	1 [ref]
Above the median ^f^	258 (50.1)	240 (51.4)	12 (35.3)	6 (42.9)	0.56 [0.30–1.04]
**Energy consumption from protein (%)**					
Below the median ^f^	260 (50.5)	240 (51.4)	11 (32.4)	9 (64.3)	1 [ref]
Above the median ^f^	255 (49.5)	227 (48.6)	23 (67.6)	5 (35.7)	1.48 [0.81–2.70]

OR: odds ratio for overweight/obesity vs. normal weight; CI: confidence interval; TV: television; CHO: carbohydrates. ^a^ In this table, continuous and categorical variables are presented as mean ± SD and *n* (%), respectively; ^b^ Lack of corresponding sum of frequencies with total sample size is due to missing data; ^c^ Children adiposity status based on the WHO 2006 BMI-for-age *z*-score cut-offs [23]; Normal weight (including at risk of overweight): −2 ≤ *z*-score ≤ +2; Overweight (not including obese): +2 < *z*-score ≤ +3; Obese: *z*-score > +3; ^d^ Crude logistic regression was conducted with the outcome variable being “overweight” and “obese”combined; ^e^ The sum of frequencies does not correspond to the total sample size given that preschoolers below the age of three years do not go to school. ^f^ Median for “total energy” corresponds to 1509 kcal; median for “energy consumption from fat” corresponds to 39.3%; median for “energy consumption from CHO” corresponds to 48.6%; median for “energy consumption from protein” corresponds to 13.15%; * The number of mothers included in this variable is 483, after exclusion of pregnant women (*n* = 42). Bolded numbers are significant at *p* < 0.05.

**Table 3:** Associations of overweight ^a^ with selected demographic, socioeconomic, parental, and dietary variables among preschoolers (*n* = 525).

Variables	Model 1 ^b^	Model 2 ^b^	Model 3 ^b^
	OR [95% CI]		
**Demographic, socioeconomic and parental variables**			
**Gender**			
Boys	1 [ref]	1 [ref]	1 [ref]
Girls	0.97 [0.52–1.83]	1.07 [0.56–2.07]	0.96 [0.49–1.88]
**Child’s age (years)**			
mean ± SD	0.99 [0.68–1.44]	0.91 [0.63–1.33]	0.92 [0.63–1.35]
**Father’s education ^c^**			
Primary or less	1 [ref]	1 [ref]	1 [ref]
Intermediate	**5.16 [1.19–22.41]**	**5.81 [1.27–26.51]**	**5.77 [1.24–26.96]**
High school and above	**5.31 [1.04–27.26]**	**5.22 [0.96–28.39]**	5.02 [1.03–27.91]
**Mother’s BMI (Kg/m^2^)**			
mean ± SD	**1.06 [1.01–1.13]**	**1.09 [1.03–1.16]**	**1.08 [1.02–1.15]**
**Presence of paid helper**			
No	1 [ref]	1 [ref]	1 [ref]
Yes	**2.19 [1.05–4.56]**	**2.34 [1.05–5.21]**	**2.30 [1.02–5.17]**
**Crowding index**			
<1 person/room	1 [ref]	1 [ref]	1 [ref]
≥1 person/room	**0.42 [0.19–0.97]**	0.47 [0.19–1.15]	**0.41 [0.17–1.02]**
**Breastfeeding history and eating habits**			
**Breastfeeding duration**	------------		
<12 months		1 [ref]	1 [ref]
≥12 months		0.62 [0.27–1.42]	0.62 [0.27–1.44]
**Eating in front of the TV**	------------	**1.07 [1.02–1.13]**	**1.08 [1.02–1.14]**
**Eating out**	------------	1.23 [0.93–1.63]	1.22 [0.92–1.62]
**Eating the same meal as the family at home**	------------	0.95 [0.89–1.01]	0.95 [0.89–1.01]
**Satiety responsiveness**	------------	**0.83 [0.70–0.99]**	**0.8 [0.68–0.99]**
**Food responsiveness**	------------	1.14 [0.87–1.49]	1.16 [0.88–1.52]
**Dietary variables**			
**Total daily energy (Kcal) ^d^**	------------	------------	
Low			1 [ref]
High			0.72 [0.35–1.50]
**Energy consumption from Fat (%) ^d,e^**	------------	------------	
Low			1 [ref]
High			**2.31 [1.13–4.75]**
**−2 Log Likelihood**	274.89	252.82	247.2
**Nagelkerke R^2^**	0.12	0.21	0.23
**Nagelkerke R^2^ difference**	0.12	0.09	0.02

OR: odds ratio; CI: confidence interval. ^a^ Overweight (including obesity) defined based on the WHO 2006 sex and age specific + 2 BMI *z*-scores [23]; ^b^ Model 1: adjusted for gender, age, father’s education, presence of paid helper, crowding index and mother’s BMI; Model 2 = Model 1 + adjustment for eating behavior variables; Model 3 = Model 2 + adjustment for dietary variables; ^c^ Low, medium and high education levels refer to primary or less, intermediate or high school and above, respectively; ^d^ Low and high total energy and energy from fat refer to first and second median, respectively; ^e^ Fat intake based on percent contribution to daily energy intake. Bolded numbers are significant at *p* < 0.05.

**Table 4:** Prevalence of overweight and obesity among Lebanese preschool children compared to those in selected countries.

Country	Date of Surveys	Criteria Used	Age (Years)	Overweight ^b^ (%)		Obesity (%)	
				Boys	Girls	Boys	Girls
WHO-2006 ^c^							
Lebanon ^a^	2010		2–5	6.4	6.6	2.8	2.5
China (Beijing) [41]	2004		2–5	4.6	2.7	2.9	1.7
Bahrain [39]	2003		2–5	9.8	10.1	7.1	5.9
Jordan [42]	2010		1–5	6.7	7.3	2.5	1.1
Qatar (Doha) [40]	2009–2010		2–5	10.6	15.2	15.5	12.5
The Netherlands [34]	2002–2006		2–5	6	4.1	5	2.9
Romania [35]	2004		2–5	5.7	4.2	2.1	2.2
Spain [43]	2006		2–5	9.6	12.2	8.8	4.4
Italy [36]	2005		2–5	5.9	5.7	4.1	2.6
Cyprus [37,38]	2004		2–5	3.3	4.7	1.8	1.3
England [44]	2002		2–5	9.8	7.5	2.5	2.2
IOTF ^d^							
Lebanon ^a^	2010		2–5	12.9	13.9	2.9	4.9
Canada [46]	2004		2–5	13	19	6	6
Australia [45]	2007		2–3	17	14	4	4
CDC ^e^							
Lebanon ^a^	2010		2–5	14.7	17.6	11.1	10.2
Iran (Tehran) [47]	2009–2010		3–6	9.8	10.3	4.8	4.5
United States of America [48]	2011–2012		2–5	23.9	21.7	9.5	7.2
Saudi Arabia (Khobar) [49]	2006		2–4	19.6	16.3	20	18.1

^a^ Current study; ^b^ Overweight (not including obesity); ^c^ WHO-2006: World Health organization 2006 reference [23]; ^d^ IOTF: International Obesity Task Force [27]; ^e^ CDC: Center for Disease Control and Prevention-2000 [25,26].
